# Scutellarein treats neuroblastoma by regulating the expression of multiple targets

**DOI:** 10.1002/ibra.12100

**Published:** 2023-05-13

**Authors:** Chen‐Yang Zhai, Ji‐Sheng Fan, Rong‐Ping Zhang

**Affiliations:** ^1^ Institute of Neuroscience Kunming Medical University Kunming China; ^2^ School of Pharmacy and Medical Sciences, Division of Health Sciences University of South Australia Adelaide Australia; ^3^ Faculty of Pharmacy Kunming Medical University Kunming Yunnan China

**Keywords:** HS‐SY5Y, neuroblastoma, proliferation inhibition, scutellarin

## Abstract

The aim of this study is to investigate the effect of scutellarein on the proliferation of neuroblastoma cells and the underlying mechanism. Six cell lines were used with drug intervention. Cell Counting Kit‐8 was used to select the best, namely, SH‐SY5Y, and then its IC_50_ value was determined. To further investigate the mechanism of scutellarin affecting SH‐SY5Y proliferation, quantitative real‐time polymerase chain reaction (qRT‐PCR) was used to detect the expression levels of 11 factors. Scutellarin administration with 300 μM significantly reduced the number of SH‐SY5Y, especially on the 3rd day of exposure to scutellarin. The IC_50_ value of scutellarin in SH‐SY5Y cells was determined to be 117.8 μM. But the practical results showed that 300 μM was the optimal concentration of scutellarin. qRT‐PCR further detected upregulated maternally expressed gene 3 (MEG3), oncogene c‐Fos (c‐FOS), and c‐jun and downregulated M2 isoform of pyruvate kinase (PKM2), non‐SMC Condensin I Complex Subunit H (NCAPH), epidermal growth factor receptor (EGFR), transforming growth factor (TGF)‐β1, and TGF‐α, suggesting that scutellarin with 300 μM volume inhibited the survival of SH‐SY5Y by regulating the expression of these 8 factors. Scutellarin could be a novel drug for the treatment of neuroblastoma, and its underlying mechanism may be related to the upregulated levels of MEG3, c‐FOS, and c‐jun and downregulated the expression of PKM2, NCAPH, EGFR, TGF‐β1, and TGF‐α.

## Graphical abstract

Cellular culture and SH‐SY5Y screening were applied as the first step, followed by administration of four different concentrations of scutellarin (0, 75, 150, and 300 μM). The morphology was continuously examined under a light field for 3 days. Subsequently, the Cell Counting Kit‐8 assay was used to determine the number of SH‐SY5Y and the IC_50_ value was also determined. Finally, molecular changes were investigated by quantitative real‐time polymerase chain reaction. In this study, baicalin inhibited the proliferation of SH‐SY5Y through multitarget regulation.

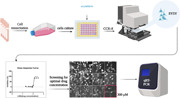

## 1 INTRODUCTION

Neuroblastoma, an embryonal tumor of the sympathetic nervous system, develops during fetal or early postnatal life from sympathetic cells arising from the neural crest. It is the most common solid extracranial malignancy in children^1^, ^2^, ^3^ and is the leading cause of cancer‐related death in children aged 1–5 years.^4^ Statistics showed that, in 2010, the age‐specific incidence in the United States was 10.7 cases per 1,000,000 persons aged 0–14 years.^5^ With the advancement of science and technology, the use of biological markers has now begun.^6^ However, these methods could not completely inhibit the recurrence and metastasis of neuroblastoma.^5^, ^7^ In addition, neuroblastoma is a highly heterogeneous disease both biologically and clinically, and its treatment depends on the disease stage, age, and biological prognostic factors.^8^, ^9^ In view of these circumstances, there is an urgent need to identify new therapeutic strategies and drugs for neuroblastoma.

**Figure 1:**
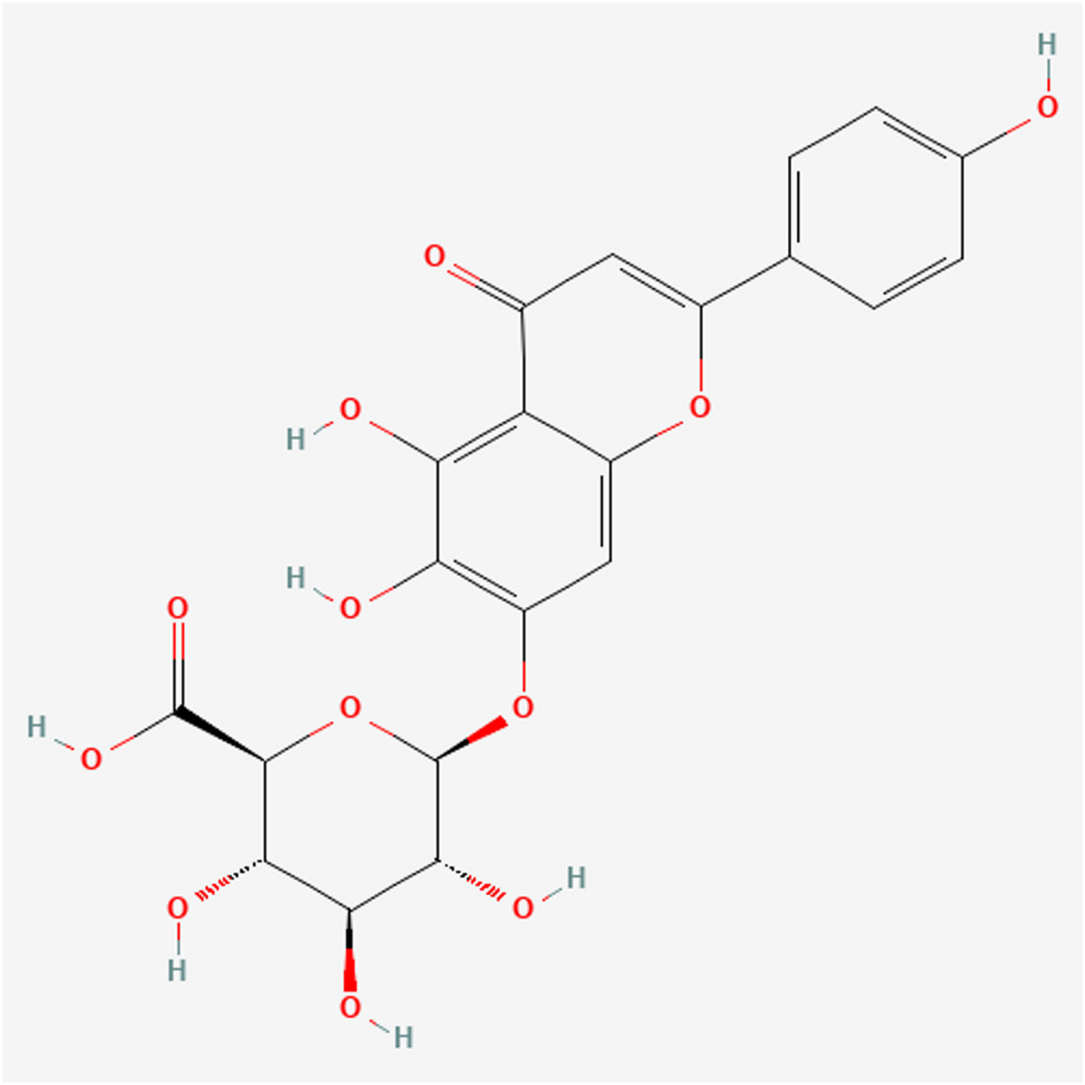
Molecular formula of scutellarin. [Color figure can be viewed at wileyonlinelibrary.com]

Scutellarin is a naturally occurring flavonoid found in *Scutellaria barbata* and *Scutellaria lateriflora*.^10^, ^11^ Its structural formula is 4,5,6‐trihydroxyflavone‐7‐glucuronide^12^ (Figure 1). Scutellarin has been shown to have a variety of beneficial biological effects, including reduced peripheral resistance, arteriole dilation, antimyocardial and cerebral ischemia,^13^, ^14^ and antioxidant, anti‐inflammatory, and antitumor activities. Its antitumor effect is worth studying.^12^ Current studies have shown that scutellarin can inhibit the metastasis of various tumors, such as hepatocellular carcinoma (HCC),^15^, ^16^ colorectal cancer,^17^ and tongue squamous cell carcinoma.^16^ In addition, scutellarin has been reported to retain chemical sensitivity, which can be demonstrated by reduction of chemical resistance in several cancer cells.^18^, ^19^ Scutellarin has also a strong M2 isoform of pyruvate kinase (PKM2) activation effect and can be used as an anticancer drug to inhibit the growth of prostate cancer cells.^20^, ^21^, ^22^ However, whether scutellarin can inhibit neuroblastoma has not been clarified as yet.

Based on the above information, scutellarin has good potential for use in cancer treatment. Existing studies mainly focused on interventions targeting proteins that are effective in experimental models, but only limited clinical efficacy has been identified. Thus, this study simulated human bone marrow neuroblastoma using SH‐SY5Y cell lines and attempted to determine the efficacy of scutellarin in treating neuroblastoma and elucidate the underlying mechanism, which may lead to the development of a new strategy for the treatment of human bone marrow neuroblastoma.

## 2 MATERIALS AND METHODS

### 2.1 Cell cultures

SH‐SY5Y was cultured in Dulbecco's modified Eagle medium (DMEM; Gibco Inc.) with 15% fetal bovine serum (FBS; Invitrogen) and antibiotics (100 U/mL penicillin and 10^−6^ μg/mL streptomycin; Gibco Inc.). A549 was grown in M‐199 medium (Gibco) supplemented with 10% FBS, 3.2 mM glutamine (Sigma), and antibiotics. AGS was incubated in Roswell Park Memorial Institute 1640 (RPMI; Gibco Inc.) supplemented with 10% FBS and antibiotics. PANC‐1 was grown in RPMI supplemented with 10% FBS and antibiotics. RKO was grown in RPMI supplemented with 10% FBS and antibiotics, and U251 was grown in RPMI supplemented with 10% FBS and antibiotics.

### 2.2 Scutellarin administration

Before scutellarin administration, cells (U251, PANC‐1, A549, AGS, RKO, and SH‐SY5Y) were cultured at a density of 8000 cells/100 μL/well in a 96‐well plate at 37°C with 5% CO_2_ for 24 h. When the degree of cell fusion reaches about 40%, the medium should be replaced. Each cultured cell line was randomly divided into five groups: control group (without scutellarin), Scu‐35 group (35 μM scutellarin), Scu‐75 group (75 μM scutellarin), Scu‐150 group (150 μM scutellarin), and Scu‐300 group (300 μM scutellarin). Three wells were assigned to each group. After 24 h of cell culture, the corresponding scutellarin ethyl solution was added and incubated at 37°C for 48 h, and the cell viability was detected using the Cell Counting Kit‐8 (CCK‐8).

### 2.3 Cell viability assay (CCK‐8)

CCK‐8 was used to detect the cell activity of cultured cells lines (U251, PANC‐1, A549, AGS, RKO, and SH‐SY5Y) after scutellarin administration and without scutellarin. [Correction added on 31 May 2024, after first online publication: The preceding sentence was revised for more clarity at the request of author.] Cells were inoculated in 96‐well plates at a density of 4000 cells/well in a total volume of 100 μL medium containing 10% FBS. Scutellarin was added to the cells 24 h after culturing. Following coincubation, cell viability was determined by CCK‐8 assays. CCK‐8 reagent was added to the wells (10 μL/well). Four hours later, optical density (OD) at 450 nm was measured. All the procedures were performed in triplicate and repeated at least three times. The inhibition ratio of scutellarin was expressed as the percentage of the control treated with vehicle solutions.

### 2.4 Detection of IC_50_

Scutellarin, consisting of yellow loose lumps and dissolved in pure water, was implanted into the previously cultured SH‐SY5Y. The drug concentration was set as 1, 2, and 3 μM, respectively, for the detection of the drug concentration of IC_50_, LogIC_50_, and Hill Slope, respectively, using CCK‐8 assays.

### 2.5 Exploration of the optimum concentration of scutellarin

After the IC_50_ of scutellarin was determined, 117.8 μM was used as the middle point, and three concentrations of 75, 150, and 300 μM up and were set up and down. Light field photographs were taken on the 1st, 2nd, and 3rd days to determine the best concentration of scutellarin.

### 2.6 Quantitative real‐time polymerase chain reaction (qRT‐PCR) analysis

To further explore the potential mechanism by which scutellarin affects the proliferation of SH‐SY5Y cell lines, in this study, qRT‐PCR was used to detect the molecular expression changes of SH‐SY5Y cell lines after scutellarin administration. Scutellarin at concentrations of 75, 150, and 300 μM were cocultured with SH‐SY5Y cells for 72 h and detected by qRT‐PCR. Total RNA of SH‐SY5Y cells was isolated using the TRIzol RNA Extraction Kit (Invitrogen), and the isolated total RNA was reverse‐transcribed to complementary DNA using a reverse transcription kit (Takara). Next, SYBR Premix Ex Taq II (Takara) was used to perform qRT‐PCR amplification. The primer sequences of oncogene c‐Fos (c‐Fos), c‐jun, epidermal growth factor (EGF), epidermal growth factor receptor (EGFR), interleukin‐6 (IL‐6), maternally expressed gene 3 (MEG3), non‐SMC Condensin I Complex Subunit H (NCAPH), PKM2, Survivin, transforming growth factor (TGF)‐α, and TGF‐β1 are shown in Table 1. The reaction system of 25 μL was prepared (Table 2), the prepared solution was tested by the machine, and the standard two‐step amplification method was used for standard amplification. The following procedures were set up for the qPCR instrument (Table 3). The relative expression of mRNA was analyzed using the $2^{-∆∆C_{t}}$ method.

**Table 1:** Primer information of c‐FOS, c‐jun, EGF, EGFR, IL‐6, MEG3, NCAPH, PKM2, Survivin, TGF‐α, and TGF‐β1.

Primer	Location	Sequence
c‐FOS	F	3′‐CACCAGGAACGAAAGTCAA‐5′
	R	3′‐CAACAACATCAGTCCCAAGA‐5′
c‐jun	F	3′‐GCAGAGCATCGGCAGAA‐5′
	R	3′‐GATTCCGGCACTTGGCT‐5′
EGF	F	3′‐GGAGCCAACCAACGTGA‐5′
	R	3′‐GTCCCCGCTTCAGTAACAA‐5′
EGFR	F	3′‐TATGTTGATGGTCCCCACT‐5′
	R	3′‐GGCAGACGTTATTGGCAT‐5′
IL‐6	F	3′‐GGCTACCATGCCAACTTCTG‐5′
	R	3′‐CGTAGTAGACGATGGGCAGT‐5′
MEG3	F	3′‐CTGCTTCCTGATGGGAAACG‐5′
	R	3′‐TCTGGCTGCATCCACCATTA‐5′
NCAPH	F	3′‐GCAATCCGGAACCAGATCAT‐5′
	R	3’‐CAGGAATTCTTCCAGCTTTCT‐5′
PKM2	F	3′‐CAGATTTGGGTTTCGTAGACA‐5′
	R	3′‐CAGACTCATTATGCGTTTCAC‐5′
Survivin	F	3′‐TCACCCTGGACAACGCCTAC‐5′
	R	3′‐TCATCAAACCTGCGGACACC‐5′
TGF‐α	F	3′‐ACCACCGCATCTCCACCTTC‐5′
	R	3′‐TTCCCAGCCTTCCAGTTCCT‐5′
TGF‐β1	F	3′‐CCTGGCTGTCCTCATTATCACC‐5′
	R	3′‐GCAGGCAGTCCTTCCTTTCA‐5′

Abbreviations: c‐FOS, oncogene c‐Fos; EGF, epidermal growth factor; EGFR, epidermal growth factor receptor; IL‐6, interleukin‐6; MEG3, maternally expressed gene 3; NCAPH, non‐SMC Condensin I Complex Subunit H; PKM2, M2 isoform of pyruvate kinase; TGF‐α, transforming growth factor‐α; TGF‐β1, transforming growth factor‐β1.

**Table 2:** Reaction system for qRT‐PCR.

Reagent	Amount used (μL)
TB Green	12.5
Forward primer	1
Reverse primer	1
cDNA	1
DDH_2_O	9.5
Amounts	25

Abbreviations: cDNA, complementary DNA; qRT‐PCR, quantitative real‐time polymerase chain reaction.

**Table 3:** Parameter setting for qRT‐PCR.

Process	Temperature (°C)	Time	Cycle number
Predegeneration	95	10 min	1
Denaturation	95	15 s	40
Annealing	60	30 s	
Elongation and fluorescence detection	72	30 s	

Abbreviation: qRT‐PCR, quantitative real‐time polymerase chain reaction.

### 2.7 Statistics

All data were expressed as mean ± standard deviation (SD). Experimental data were compared using one‐way analysis of variance (ANOVA) with SPSS17.0 software. Data were first tested for normal distribution; one‐way ANOVA was performed for a normal distribution, and a nonparametric log‐rank test was used for a non‐normal distribution. Statistical significance was accepted at *p < 0.05.

## 3 RESULTS

### 3.1 Effects of scutellarin on various tumor cell lines

**Figure 2:**
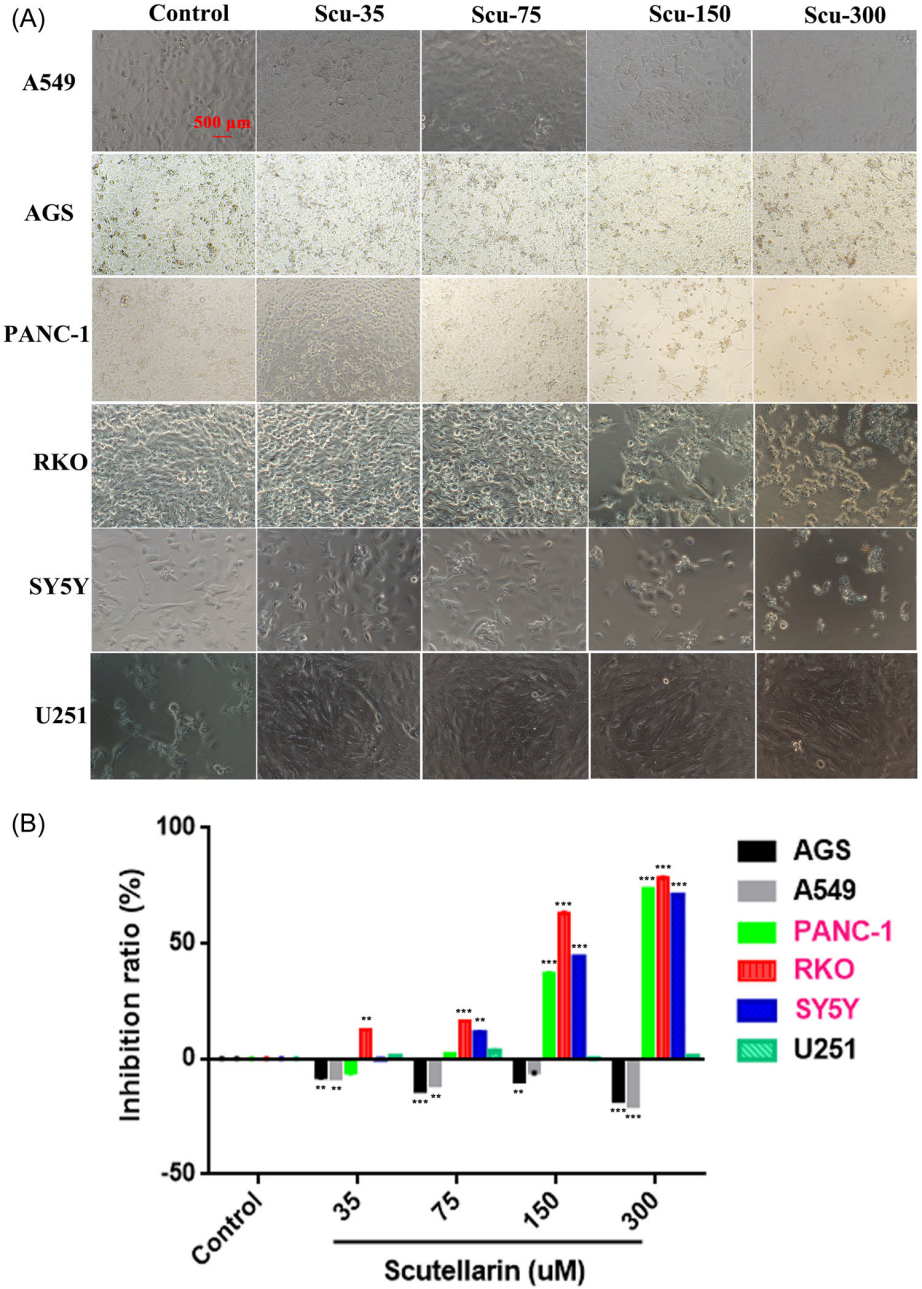
Cellular screening. (A) Morphology of six alternative cell lines in five groups with different drug dosages (Control, Scu‐35, Scu‐75, Scu‐150, and Scu‐300) under the light field. (B) The bar chart shows the inhibition ratio of each cell line in each group by Cell Counting Kit‐8 (*p < 0.05) (Control: no scutellarin; Scu‐75: add 75 μM scutellarin; Scu‐150: add 150 μM scutellarin; and Scu‐300: add 300 μM scutellarin). [Color figure can be viewed at wileyonlinelibrary.com]

Six cell lines were recruited, and each cell line was divided into five groups (Control, Scu‐35, Scu‐75, Scu‐150, and Scu‐300 groups) with different drug dosages separately. [Correction added on 31 May 2024, after first online publication: In the preceding sentence the word “Scu‐50” was revised to “Scu‐35” for clarity at the request of author.] Light field images showed that all cells in the Scu‐300 group showed the fewest cellular number and the lowest cellular density when compared with other groups individually (Figure 2A). CCK‐8 analysis revealed that the more the drug dosage in each group, the higher the inhibition ratio. The figure shows that scutellarin has an inhibitory effect on RKO, PANC‐1, and SH‐SY5Y (*p* < 0.05, Figure 2B).

### 3.2 The exploration of SH‐SY5Y's IC_50_

**Figure 3:**
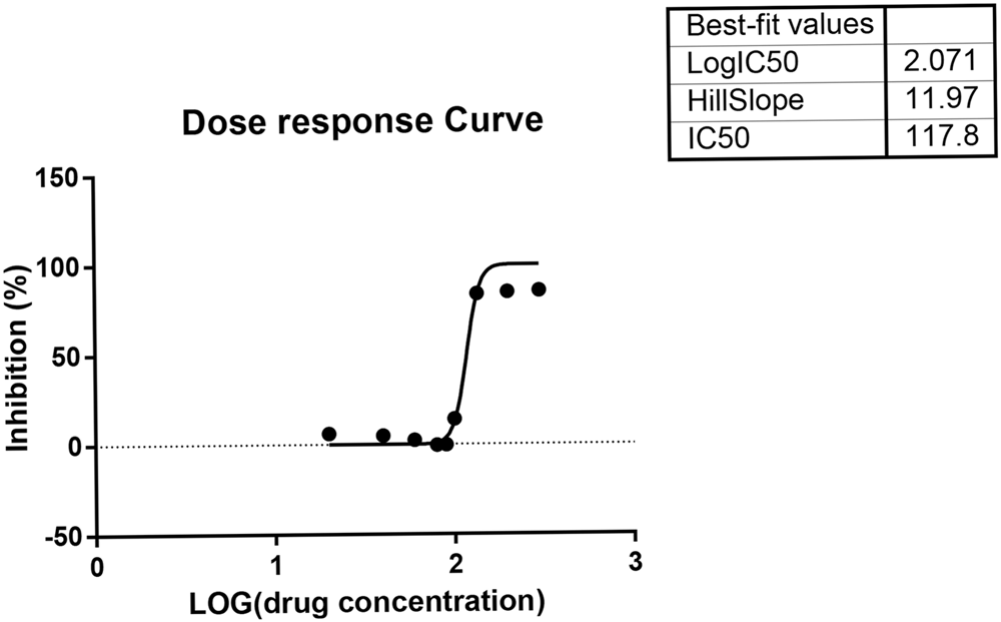
The best‐fit values of scutellarin concentration. The curve chart reveals the dose–response curve following medication. The table in the top right corner provides the best‐fit values of IC_50_.

Whether scutellarein can inhibit neuroblastoma is not clear, but the results of this study showed that scutellarein has an inhibitory effect on SH‐SY5Y. The human cell line SH‐SY5Y is commonly used as an in vitro model of human neurons and as a model for neuronal function and differentiation in neuroscience studies.^23^, ^24^ Specifically, SH‐SY5Y cells have been used to study neuroprotection, neurotoxicity, and neuroblastoma tumor genesis.^25^, ^26^ Therefore, in this study, the aim is to conduct further experiments to detect SH‐SY5Y differentially expressed genes after baicalein treatment to explore the potential inhibitory mechanism of scutellarin. To determine the best hemi‐inhibitory concentration, CCK‐8 assays were performed to detect the cell viability of SH‐SY5Y after scutellarin treatment. IC_50_ value of scutellarin in SH‐SY5Y cells was determined to be 117.8 μM at 48th hour (Figure 3).

### 3.3 Search for the optimal concentration of drugs

**Figure 4:**
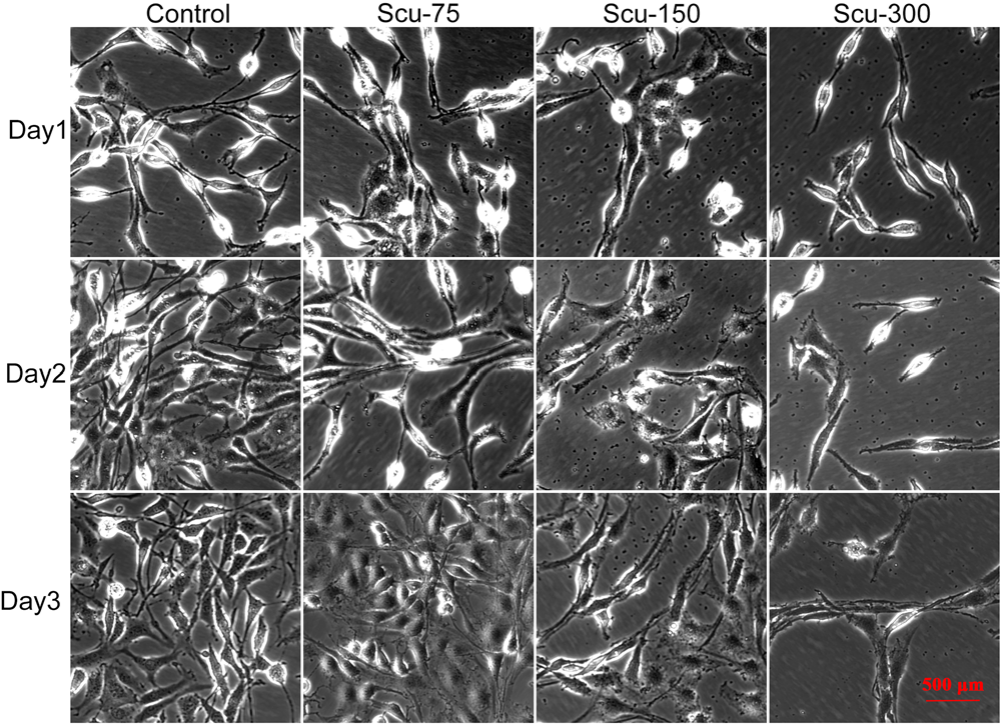
Morphology of SH‐SY5Y after scutellarin administration. SH‐SY5Y were divided into four groups (Control: no scutellarin; Scu‐75: Add 75 μM scutellarin; Scu‐150: Add 150 μM scutellarin; and Scu‐300: Add 300 μM scutellarin) and were cultured for 3 days. The morphology in each group of each day was visible under the light field. [Color figure can be viewed at wileyonlinelibrary.com]

According to the results of IC_50_, three gradients with IC_50_ as the intermediate value were set in this study, which were 75, 150, and 300 μM, respectively. The light field revealed a gradual decrease in the number of cells from the Control group to the Scu‐300 group. Besides, SH‐SY5Y showed morphological impairment with the smallest cellular size and shortest neurites in the Scu‐300 group. Meanwhile, on the 3rd day of culturing in the Control group, Scu‐75 and Scu‐150 groups showed the maximum density and number of SH‐SY5Y cells, as compared with that of the 1st and 2nd days, while the Scu‐300 group revealed the least number of SH‐SY5Y on the 3rd day (Figure 4).

### 3.4 Upregulated level of MEG3 and downregulated levels of PKM2, NCAPH, and TGF‐α following scutellarin administration

**Figure 5:**
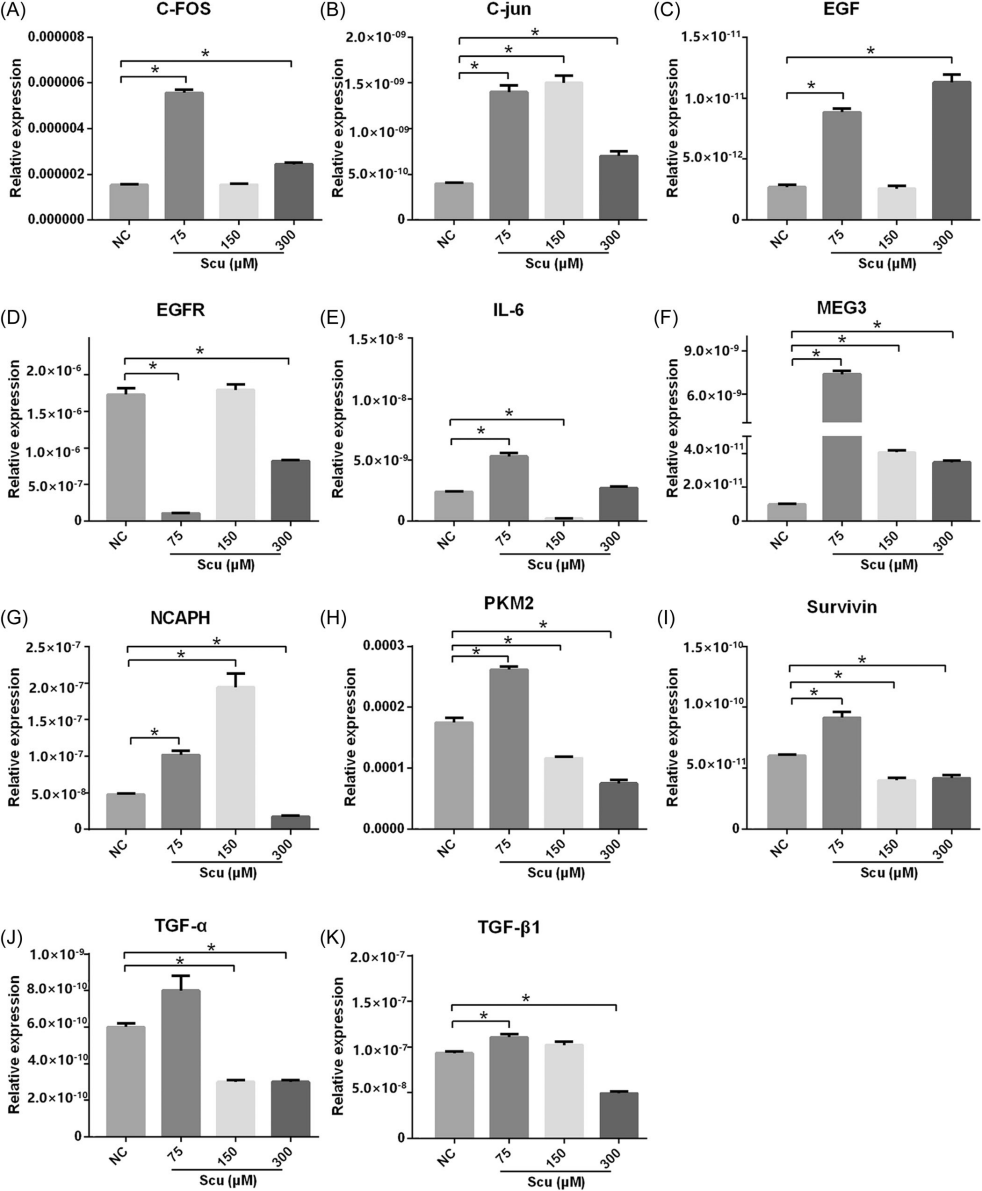
Molecular changes following scutellarin administration. The bar charts (A–K) reveal the changes in the relative expressions of c‐FOS, c‐jun, EGF, EGFR, IL‐6, MEG3, NCAPH, PKM2, Survivin, TGF‐α, and TGF‐β1 driven by scutellarin administration (A–K). c‐Fos, oncogene c‐Fos; EGF, epidermal growth factor; EGFR, epidermal growth factor receptor; IL‐6, interleukin‐6; MEG3, maternally expressed gene 3; NCAPH, non‐SMC Condensin I Complex Subunit H; PKM2, M2 isoform of pyruvate kinase; TGF‐α, transforming growth factor‐α; TGF‐β1, transforming growth factor‐β1.

Previous results showed that 300 μM scutellarin had the best inhibitory effect on cell growth, so our molecular study focused on the expression state at 300 μM. To explore the potential mechanism by which scutellarin affects the proliferation of SH‐SY5Y cell lines, qRT‐PCR was used to determine mRNA levels of c‐FOS, c‐jun, EGF, EGFR, IL‐6, MEG3, NCAPH, PKM2, Survivin, TGF‐α, and TGF‐β1. The results showed that the expression of c‐FOS was upregulated in the Scu‐75 group and the Scu‐300 group (*p* < 0.05, Figure 5A). The expression of c‐jun was upregulated in the Scu‐75 group, the Scu‐150 group, and the Scu‐300 group (*p* < 0.05, Figure 5B). The expression trend of EGF was consistent with that of c‐FOS (*p* < 0.05, Figure 5C). The expression of EGFR was downregulated in the Scu‐75 group and the Scu‐300 group (*p* < 0.05, Figure 5D). The expression of IL‐6 was upregulated in the Scu‐75 group but was downregulated in the Scu‐150 group (*p* < 0.05, Figure 5E). The results showed that the expression of MEG3 was considerably upregulated in the Scu‐75 group but slightly increased in the Scu‐150 and Scu‐300 groups, as compared with the NC group (*p* < 0.05, Figure 5F). Similarly, the level of NCAPH was increased in the Scu‐75 and Scu‐150 groups and lconsiderably decreased in the Scu‐300 group compared with the NC group (*p* < 0.05, Figure 5G). However, the expression of PKM2 was upregulated in the Scu‐75 group and downregulated in the Scu‐150 and Scu‐300 groups compared with the NC group (*p* < 0.05, Figure 5H). The expression of Survivin was upregulated in the Scu‐75 group but was downregulated in the Scu‐150 group and the Scu‐300 group (Figure 5I). The level of TGF‐α was decreased in both Scu‐150 and Scu‐300 groups, as compared with the NC group (*p* < 0.05, Figure 5J), but the level of TGF‐β1 was increased in the Scu‐75 group and decreased in the Scu‐300 group (*p* < 0.05, Figure 5K).

## 4 DISCUSSION

In the present study, the SH‐SY5Y cell line was recruited after cellular screening of six cell lines: SH‐SY5Y, A549, AGS, PANC‐1, RKO, and U251. A subsequent study found that a drug concentration of 300 μM of scutellarin could best inhibit the survival of SH‐SY5Y. Besides, SH‐SY5Y showed morphological impairment, with a small cell size and short neurites. Further study found the possible mechanism related to the upregulation of MEG3, c‐FOS, and c‐jun and downregulation of the expression of PKM2, NCAPH, EGFR, TGF‐β1, and TGF‐α.

### 4.1 Effects of scutellarin on various tumor cell lines

Scutellarin has been shown to inhibit the proliferation, invasion, and migration and promote apoptosis of gastric cancer cells. More importantly, scutellarin had no significant adverse effect on body weight gain and serum biochemical parameters in mice.^27^ In this study, we selected SH‐SY5Y cells for exploration. SH‐SY5Y, neuroblastoma, a human‐derived cell line used in scientific research and one of the most common types of pediatric tumors that could spread quickly in neuronal tissues, was cloned from a bone marrow biopsy‐derived line called SK‐N‐SH and first reported in 1973.^28^ SH‐SY5Y cells can migrate by mitosis and differentiate by extending neurites to surrounding areas, spontaneously transforming into neuroblast‐like cells and epithelioid cells in vitro. The dividing cells could form clusters of cells that were reminders of their cancerous nature, but certain molecules such as retinoic acid, brain‐derived neurotrophic factor (BDNF), or tissue‐type plasminogen activator (TPA) could inhibit the cells from proliferating and differentiating. Juniper berry extract induced p53‐associated apoptosis in human neuroblastoma SH‐SY5Y cells through the potentiation and synergism of several phenolic compounds.^29^ Pingping Xu et al.^30^ proved that indirubin can initiate the apoptosis process of SH‐SY5Y neuroblastoma cells by inducing G1 phase stagnation in SH‐SY5Y cells. However, it is unclear whether there are other drugs that can promote apoptosis or inhibit the growth of SH‐SY5Y. In the present study, it was found that on the same day, the group with higher scutellarin doses showed fewer number of SH‐SY5Y. However, the longer SH‐SY5Y was cultured, the lower the density of cells was when groups cultured on the 3rd day compared with the second and 1st day, indicating that scutellarin inhibited the growth of SH‐SY5Y. The human cell line SH‐SY5Y is commonly used as an in vitro model of human neurons and as a model for neuronal function and differentiation in neuroscience studies.^23^, ^24^ Specifically, SH‐SY5Y cells have been used to study neuroprotection, neurotoxicity, and neuroblastoma tumor genesis.^25^, ^26^ Therefore, in this study, the aim w asto conduct further experiments to detect SH‐SY5Y differentially expressed genes after scutellarin treatment to explore the potential inhibitory mechanism of scutellarin.

### 4.2 Scutellarin inhibited the survival of SH‐SY5Y by modulating expression of MEG3, c‐FOS, c‐jun, PKM2, NCAPH, EGFR, TGF‐β1, and TGF‐α

In the present study, it was found that the administration of 300 μM of scutellarin could be the best antagonist against SH‐SY5Y. Consistently, upregulated levels of MEG3, c‐FOS, and c‐jun and downregulation of the expression of PKM2, NCAPH, EGFR, TGF‐β1, and TGF‐α were detected following scutellarin administration with 300 μM volume.

C‐fos and c‐Jun are transcription factors. c‐fos is a marker of neuronal activation and plays an important role in neuronal activation, synaptic plasticity, and neuronal apoptosis.^31^ In response to extracellular stimuli, c‐fos is rapidly induced to cause transcription and forms a heterodimer with c‐Jun protein (transcription factor activator protein 1, AP‐1).^32^ c‐fos has been reported to promote the transcription of viral genes by directly binding to multiple promoters, thereby inducing sustained activation of extracellular signal‐regulated kinase (ERK)–mitogen‐activated protein kinase (MAPK), a signaling pathway closely related to cell proliferation.^33^ The specific role of these pathways in the proliferation response of SH‐SY5Y inhibited by scutellarein remains unclear. Our findings demonstrate the effects of prolonged, continuous baicalein exposure on intracellular signaling in SH‐SY5Y, which may be more relevant to SH‐SY5Y patients exposed to steady‐state drug levels, and suggest that the observed changes in the signaling pathway activity may also represent a mechanism of drug resistance.

MEG3, a maternally expressed, imprinted long noncoding RNA gene, was not expressed in functioning pituitary tumors and various human cancer cell lines, while its ectopic expression inhibited growth in human cancer cells, including HeLa, MCF‐7, and H4. Moreover, MEG3 was located on chromosome 14q32.3, a site that has been predicted to contain a tumor suppressor gene involved in the pathogenesis of meningiomas.^34^, ^35^ Downregulation of lncRNA MEG3 inhibited cell migration and proliferation in patients with Hirschsprung's disease.^36^ However, whether it can inhibit SH‐SY5Y cells is still unknown, which was confirmed in this experiment, namely, the upregulation of MEG3 significantly inhibits the survival of SH‐SY5Y.

TGF‐α and TGF‐β are transforming growth factors. Previous studies have shown that TGF‐α stimulated the growth of glioma cell line U251 and partially compensated for the inhibitory effect of the Notch signaling inhibitor DAPT (a highly potent inhibitor of γ‐secretase).^37^ Autosecretory TGF‐α stimulation does lead to increased tumor growth in vivo, regulated through the TGF‐α/EGFR autocrine circuit, which can be accessed.^38^ This is consistent with the results of the present study showing that EGFR was decreased after scutellarein treatment, suggesting that the effect may be achieved through the TGF‐α/EGFR autocrine circuit. Interestingly, our results showed that EGF was upregulated, while previous studies showed that EGF upregulation promoted tumor cell proliferation or invasion, and the knockdown of this gene inhibited tumor cell proliferation. This may be an apparent consequence of the decrease in EGFR after scutellarein treatment. In addition, targeted therapy of EGFR/ErbB2 inhibits ErbB2 phosphorylation and Survivin upregulation, as well as downstream ERK1/2 and AKT activation, thereby reducing neurofibroma cell proliferation,^39^ which is consistent with our results of Survivin downregulation. This indicates that scutellarein can be exploited as an EGFR inhibitor in the future.

PKM2 was a crucial regulator of the metabolic fate of the glycolytic intermediates and had the unique capacity to shift glucose metabolism in favor of cancer cells.^40^, ^41^, ^42^ Coherently, cancerous state correlated with high PKM2 expression in various tumor tissues and cell lines.^43^ Correspondently, nuclear translocation of PKM2 induced by somatostatin analogs was linked to apoptosis long before documentation of the tumor‐promoting results of nuclear PKM2.^44^, ^45^ The results of the present study showed that baicalein downregulated PKM2, which in turn inhibited the proliferation of SH‐SY5Y cells.

NCAPH, a member of the Barr family, is located in 2q11.2.^46^ There are a lot of studies in tumors that might play a crucial role in the occurrence of development and process of HCC^47^ and may also be an important contributor to the development of nonsmall cell lung cancer, that is, NCAPH can promote the proliferation of cancer cells.^48^ The knockdown of NCAPH has also been shown to reduce breast cancer cell proliferation.^49^ NCAPH promotes cell proliferation and apoptosis of bladder cancer cells through the MEK/ERK signaling pathway.^50^ These series of studies are in the same direction as our results.

**Figure 6:**
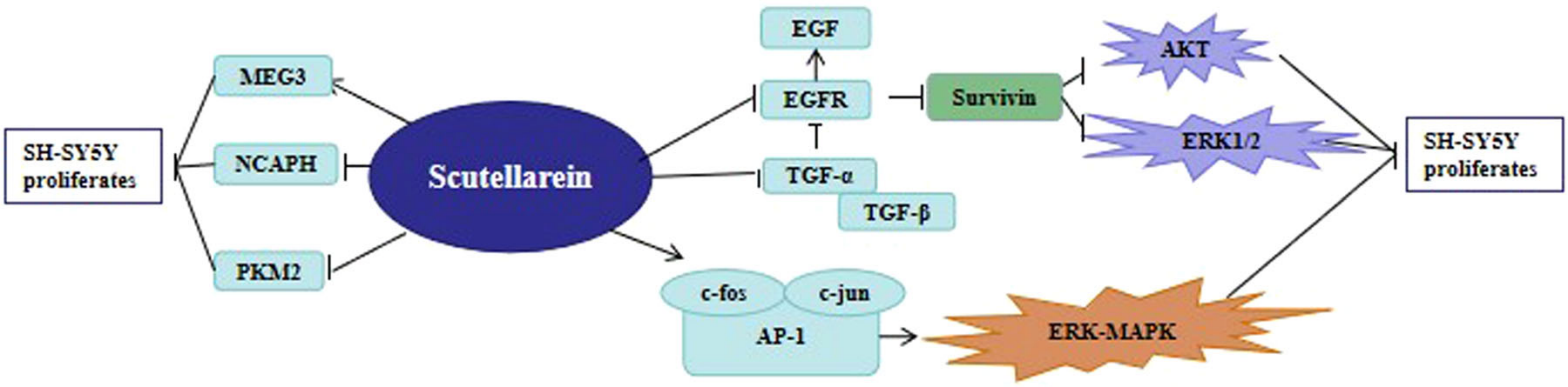
Scutellarein regulates the proliferation of SH‐SY5Y by multiple targets. [Color figure can be viewed at wileyonlinelibrary.com]

Taken together, 300 μM scutellarin, the best dose concentration in our experiment, significantly suppressed the survival of SH‐SY5Y by upregulating the expression of MEG3, c‐FOS, and c‐jun and downregulating the expression of PKM2, NCAPH, EGFR, TGF‐β1, and TGF‐α. This is the result of comprehensive regulation of multiple targets (Figure 6). This represents a new approach for the development of targeted drugs for HS‐SY5Y. At present, the specific pathways by which scutellarein regulates these 11 factors are still unclear. Therefore, future research must further clarify the exact mechanism, which remains unclear.

## AUTHOR CONTRIBUTIONS

Chen‐Yang Zhai conducted the experiments and wrote the preliminary manuscript. Ji‐Sheng Fan analyzed data, and Rong‐Ping Zhang revised the final manuscript; All the authors have read and approved the final manuscript as submitted.

## CONFLICT OF INTEREST STATEMENT

The authors declare no conflict of interest.

## ETHICS STATEMENT

All the cell lines were donated from Jia Liu from Kunming Medical University and this study does not involve experimental animals and human subjects, therefore, it does not involve ethics.

## Data Availability

The data within this article is available and can be obtained on request.
